# Effects and Interaction of Icariin, Curculigoside, and Berberine in Er-Xian Decoction, a Traditional Chinese Medicinal Formula, on Osteoclastic Bone Resorption

**DOI:** 10.1155/2012/490843

**Published:** 2012-11-01

**Authors:** Liming Xue, Lei Jiao, Yin Wang, Yan Nie, Ting Han, Yiping Jiang, Khalid Rahman, Qiaoyan Zhang, Luping Qin

**Affiliations:** ^1^School of Pharmacy, Second Military Medical University, 325 Guohe Road, Shanghai 200433, China; ^2^Department of Pharmaceutical, PLA 455 Hospital, Shanghai 200052, China; ^3^School of Pharmacy and Biomolecular Sciences, Liverpool John Moores University, Byrom Street, Liverpool L3 3AF, UK

## Abstract

Er-Xian decoction (EXD), a traditional Chinese medicine, has been reported to have a protective effect against bone loss in ovariectomized osteoporotic rats, and the inclusion of icariin (I), curculigoside (C), and berberine (B) in EXD displays inhibitory effects on osteoclastic bone resorption. In the present paper, we investigated the interaction and effects of I, C, B, and their combination on bone resorption activity *in vitro* on osteoclasts derived from rat bone marrow cells. ICB synergistically decreased the formation of bone resorption pits, the number of multinucleated osteoclasts, and the activity of tartrate-resistant acid phosphatase (TRAP) and showed antagonistic or additive effects on cathepsin K activity in the coculture system of osteoblasts and bone marrow cells in the presence of 1, 25-dihydroxyvitamin D_3_ and dexamethasone. The combination of ICB also enhanced the inhibitory effects on the formation of F-actin ring, a cytoskeleton structure of osteoclasts induced from bone marrow cells with macrophage colony stimulation factor (M-CSF) and receptor activator of NF-**κ**B ligand (RANKL). In addition, ICB synergistically improved the ratio of protein expression of osteoprotegerin (OPG) and RANKL in osteoblasts and interfered with the mitogen-activated protein kinases (MAPKs) pathway in osteoclast. These results clearly show that I, C, B, and their combination in EXD exert effects of mutual reinforcement. However, IBC does not show an intensified adverse effect in the ovariectomized murine model, as revealed by change in body and uterine weight, confirming the safety of EXD. These observations are in agreement with the rationality of the formula used in this paper.

## 1. Introduction 

Traditional Chinese medicine (TCM), an empirical system of multicomponent therapeutics, potentially meets the demands of treating a number of complex diseases in an integrated manner, in particular chronic diseases and metabolic syndromes [1]. Naturally occurring herbs and herbal ingredients organized into certain formula have been shown to have potential interaction effects, including mutual enhancement, mutual assistance, mutual restraint, and mutual antagonism. For example, synergistic interactions occur when the efficacy of combinations of herbs (or ingredients) is greater than the summed responses of each individual herb or ingredient [2, 3]. Scientific evidence for TCM is generally achieved through rigorous experimental design, which has been dominated by the search for its biological basis, identification of active substances, and the investigation of the mechanisms of its action. In contrast, research in the role and interaction of active ingredients in the formulas is still scarce, thus hampering the understanding of the rationality of formula design in TCM.

Postmenopausal osteoporosis is believed to be associated with ovarian hormone deficiency and is by far the most common cause of age-related bone loss [4]; and with the reduction in estrogen levels, there is an increase in bone breakdown relative to bone formation, microarchitectural deterioration, and decreased bone mass [4, 5]. The excessive bone breakdown is caused by an increased number of osteoclast and enhanced ability of bone resorption [6]. Osteoclasts are multinucleated cells (3–10 nuclei per cell) differentiated from hematopoietic precursors and are involved in bone remodeling. Through osteoclastic bone resorption, osteoclasts play both a crucial physiological role in bone remodeling and a pathological role in osteoporosis as characterized by excess activity of osteoclasts [6, 7].

The osteoclasts are formed and differentiated under the control of some cytokines, such as macrophage colony stimulation factor (M-CSF), receptor activator of NF-*κ*B ligand (RANKL), and osteoprotegerin (OPG) [7, 8]. The binding of RANKL to its receptor RANK leads to recruitment of TNF receptor-associated factor 6 (TRAF6) to the cytoplasmic domain of RANK. This then activates the downstream targets of TRAF6 including transcription factors such as nuclear factor kappa B (NF-*κ*B), activator protein-1 (AP-1), and nuclear factor of activated T cells (NFAT), as well as the mitogen-activated protein kinases (MAPKs) including p38 MAP kinases, c-Jun N-terminal kinases (JNK/SAPKs), extracellular signal-regulated kinases (ERKs), and phosphatidyl-inositol-3-kinase (PI3 K)/Akt [9–12]. Osteoprotegerin (OPG), a soluble decoy receptor of RANKL, negatively regulates osteoclast differentiation and bone resorption. Mature osteoclast formation is also associated with the expression of differentiation markers, including tartrate resistant acid phosphatase (TRAP) and cathepsin K. The modulation of these differentiation-related signaling pathways has the potential of being considered as a therapeutic strategy for the treatment of skeletal diseases [13]. 

Over the past 50 years, Er-Xian decoction (EXD), a traditional Chinese medicinal formula, has been used for the treatment of osteoporosis disorders, menopausal syndrome, and aging diseases [14, 15]. The components of EXD are Epimedii Folium (Yinyanghuo), Curculiginis Rhizoma (Xianmao), Anemarrhenae Rhizoma (Zhimu), Phellodendri Chinensis Cortex (Huangbo), Morindae Officinalis Radix (Bajitian), and Angelicae Sinensis Radix (Danggui), with icariin, curculigoside, timosaponin BII, berberin, nystose, and ferulic acid as major active ingredients [16]. According to traditional Chinese medicine, Epimedium Folium and Phellodendri Chinensis Cortex are essential ingredients of EXD and appear to play important roles in ameliorating signs and symptoms of the menopausal syndrome and osteoporosis. Curculiginis Rhizoma and Morindae Officinalis Radix help strengthen the curative effect of the Epimedium Folium, and Anemarrhenae Rhizoma helps Phellodendri Chinensis Cortex to play a fuller role. Angelicae Sinensis Radix cooperates with the above five medications to strengthen the therapeutic effects and treat the accompanying disease or syndromes [17]. We tested the effects of major active ingredients on osteoblast and osteoclast and found that icariin, timosaponin BII, nystose, and ferulic acid could increase the proliferation and alkaline phosphatase (ALP) activity of osteoblast, and icariin (I), curculigoside (C), and berberine (B) could decrease the number of TRAP positive multinucleated osteoclasts and TRAP-activity of osteoclast, as evidenced by their inhibitory effects on bone resorption [16]. 

Therefore, in the present study, we firstly proved that the combination of ICB has more potent antiosteoporotic activity than individual compounds on ovariectomized osteoporotic mice, and furthermore, we investigated the inhibitory effects of I, C, B, and their combination on bone resorption of octeoclast to evaluate the interaction between them so as to obtain an understanding of their mechanism of action and to provide an insight into the rationality of formula design for potential use in TCM.

## 2. Materials and Methods

### 2.1. Reagents

Reagents used in this study included *α*-modified minimum essential medium (*α*-MEM) and fetal bovine serum (FBS) (Gibco, US); 1, 25-dihydroxyvitamin D_3_, dexamethasone, trypsin, and coomassie brilliant blue G-250 (Sigma, US); recombinant rat M-CSF (400-28) and RANKL (400-30) (Peprotech EC, US); OPG (sc11383), RANKL (sc9073), and *β*-actin (sc81178) antibody (Santa Cruz, USA); anti-phospho-Akt (9275), anti-phospho-ERK (3371), anti-phospho-JNK (9251), anti-phospho-p38 (9211), and anti-phospho-I-*κ*B (9245) (Cell Signaling Technology, Beverly, MA); NucBuster Protein Extraction kit (Merck, Novagen, Germany); Cathepsin K activity kit (Biovision, USA); pararosaniline, Hoechst 33258, and rhodamine-conjugated phalloidin (Sigma, USA). Diolamine, potassium sodium tartrate, disodium 4-nitrophenylphosphate, Triton X-100, and 4-nitrophenol were of domestic AR grade. 

Icariin (I), curculigoside (C), and berberine (B) were isolated from Epimedii Folium, Curculiginis Rhizoma, and Phellodendri Chinensis Cortex, respectively, and were identified by spectrum analysis. The data of proton nuclear magnetic resonance spectroscopy (^1^H NMR) and mass spectrometry (MS) were coincidence with previous studies. The purities of icariin, curculigoside, and berberine were up to 99% according to high-performance liquid chromatography (HPLC) analysis.

### 2.2. Animal Experimental Protocol

Seventy 3-month-old female ICR mice were purchased from SLACOM experimental animal company (Shanghai, China) and acclimatized to laboratory conditions for 1 week before commencing the experiment. Food and drinking water were supplied *ad libitum*. The mice were weighed weekly during the experimental period. An osteoporotic model was established 12 weeks after bilateral ovariectomy. Knowing that the osteoporotic model induced by ovariectomy is associated with estrogen deficiency, nylestriol (an estrogenic substance) was used as the positive drug. Of the 70 female mice, 10 mice were sham-operated and treated with deionized water as the control group. The remaining 60 mice were bilaterally ovariectomized and equally randomized into six groups, with intragastric administration of either deionized water as the OVX model control, nylestriol (1 mg/kg) weekly as the positive control, icariin (40 mg/kg) daily, curculigoside (20 mg/kg) daily, berberine (120 mg/kg) daily, or a combination of icariin (40 mg/kg), curculigoside (20 mg/kg), and berberine (120 mg/kg) daily as the treatment groups, all for 12 weeks. Mice received treatments starting from day one after surgeries and the success of ovariectomy was confirmed at necropsy by failure to detect ovarian tissue and by observation of marked atrophy of uterine horns. At the end of the treatment, blood samples from all the mice were withdrawn by orbital sinus method and centrifuged to collect serum for the measurement of biochemical parameters. The uterine was removed and immediately weighed. 

The tibia was cleaned by removing adhering soft tissues and stored in 75% ethanol for a week prior to analysis. The bone mineral density (BMD) and bone mineral content (BMC) were measured at 3 mm from the proximal epiphysis of right tibia with a peripheral quantitative computed tomography (*p*QCT) densitometry (Stratec XCT Research SA, Germany). Serum alkaline phosphatase (ALP), tartrate-resistant acid phosphatase (TRAP), and serum creatinine (Cr) were measured on an automatic analyzer (Ciba-Corning 550, USA) using diagnostic reagent kit determination. Serum osteoprotegerin (OPG) and deoxypyridinoline cross-links (DPD) were estimated using an Elisa kit according to manufacturer instructions. The experimental protocol used in this study was approved by the Bioethic Committee of the Second Military Medical University, and the procedures employed were strictly according to generally accepted international rules and regulations.

### 2.3. The Assay of Formation, Differentiation, and Bone Resorption of Osteoclast

#### 2.3.1. Osteoclasts Induced with 1, 25-Dihydroxyvitamin *D*_3_ and Dexamethasone from Bone Marrow Cells in a Coculture System with Osteoblasts

Primary osteoblastic cells were prepared from neonatal rat calvarial osteoblasts according to the literature [18]. Induction and culture of osteoclasts were as follows. Briefly, the femur was disarticulated from Wistar rats aged 3 days, and the ends were removed, and the bone marrow cells were flushed out using a 1 mL syringe. Primary osteoblastic cells (1 × 10^5^/mL) and bone marrow cells (1 × 10^6^/mL) were cocultured in *α*-MEM medium containing 10% FBS, 1, 25-dihydroxyvitamin D_3_ (10 nmol/L) and dexamethasone (100 nmol/L) at 37°C in a humidified atmosphere of 5% CO_2_. Prior to plating the cells, cover glasses (5 × 5 mm) or dental slices (40 *μ*m thick) were placed into culture dishes. The formations of multinucleated osteoclasts (MNCs) were confirmed by TRAP staining and resorption pits formed on dental slices. Osteoclastic cells were seeded onto culture plates in *α*-MEM medium. The medium was replaced the following day with fresh medium containing 1.0 *μ*M icariin, 1.0 *μ*M curculigoside, 1.0 *μ*M berberine, or their combination (including 1.0 *μ*M icariin, 1.0 *μ*M curculigoside, and 1.0 *μ*M berberine) for indicated time. All cultures were maintained at 37°C in a humidified atmosphere of 5% CO_2_ throughout. The osteoclasts were used to investigate formation of osteoclast, TRAP and Cathepsin K activity, and bone resorption activity.

#### 2.3.2. Counting of TRAP-Positive Multinucleated Osteoclasts

Primary osteoblasts and bone marrow cells were placed on 96-well plates containing cover glasses and cultured in *α*-MEM medium containing 10% FBS, 1, 25-dihydroxyvitamin D_3_ (10 nM) and dexamethasone (100 nM), for 24 h. Cells were then treated with or without I, C, B, and their combination for 10 days and stained for TRAP with a TRAP staining kit (no. 387A-1KT; Sigma-Aldrich). Cells with three or more nuclei were counted as osteoclast-like multinucleated osteoclasts under a microscope.

#### 2.3.3. Assay for TRAP Activity

Primary osteoblasts and bone marrow cells in *α*-MEM medium containing 10% FBS, 1, 25-dihydroxyvitamin D_3_ (10 nM), and dexamethasone (100 nM) were placed in a 96-well culture dishes, cultured for 6 days, and then treated with or without I, C, B, and their combination for 48 h. TRAP was determined as follows: cells were washed twice with PBS, and then 20 *μ*L 0.1% Triton X-100 was added to the cells to induce lysis at room temperature. After 15 min, 100 *μ*L substrate solution (0.4 g disodium 4-nitrophenylphosphate and 2.0 g potassium tartrate dissolved in 200 mL of deionized water, with a pH adjusted to 3.5 with 1 mol/L HCl) was added to the lysed cells and incubated at 37°C for 30 min, the reaction was terminated by the addition of 100 *μ*L 1 mol/L NaOH to each well, and the absorbance was measured at 405 nm. At the same time, positive cells for TRAP were counted and the TRAP activity was expressed as nanomoles *p*-nitrophenol per minute per 100 osteoclasts.

#### 2.3.4. Determination of Cathepsin K Activity

Primary osteoblasts (2 × 10^6^) and bone marrow cells (2 × 10^7^) in *α*-MEM medium containing 10% FBS, 1, 25-dihydroxyvitamin D_3_ (10 nM), and dexamethasone (100 nM) were placed in a 24-well culture plate, cultured for 7 days, and then treated with or without I, C, B, and their combination for 48 h. Cells were collected by centrifugation, lysed in 50 *μ*L chilled CK cell lysis buffer, incubated on ice for 10 min, and then vortexed for 5 min. CK reaction buffer (50 *μ*L) and 10 mM CK substrate Ac-LR-AFC (2 *μ*L) were then added to the sample and the mixture solution was incubated at 37°C for 2 h. The samples were then transferred onto a 96-well plate; the intensity of fluoresce was measured in a fluorometer equipped with a 400 nm excitation filter and a 505 nm emission filter.

#### 2.3.5. Determination of Bone Resorption Pit

The primary osteoblast and bone marrow cell suspension were seeded into the wells of 96-well culture plates with a sterilized bone slice (40 *μ*m thick, 5 mm × 5 mm). After 24 h culture in *α*-MEM medium containing 10% FBS, 1, 25-dihydroxyvitamin D_3_ (10 nM), and dexamethasone (100 nM), cells were treated with or without I, C, B, and their combination for 12 days. Dental slices were treated with ultrasonic waves in 1 mol/L NH_4_OH to remove adherent cells and stained with 0.1% toluidine blue solution. Resorption pits were observed under a microscope; 20 vision fields of each dental slice were randomly chosen to measure the pit area with image analysis software (Leica Q550IW, Germany). The sum of the resorption pit area of 20 random vision fields was calculated as resorption pit area of each dental slice. The inhibitory effects of the tested compounds on bone resorption were expressed as the resorption pit area of dental slice treated with test compounds/resorption pit area of control × 100%.

### 2.4. Assay for Cytoskeleton of Osteoclasts

#### 2.4.1. Osteoclasts Induced with M-CSF and RANKL from Bone Marrow Cells

5 × 10^7^ bone marrow cells were cultured in a 6-well plate containing *α*-MEM supplemented with 10% FBS and 5 ng/mL M-CSF in a humidified atmosphere of 5% CO_2_ for 24 hours. Nonadherent cells were collected and cultured in *α*-MEM medium containing 10% FBS and 50 ng/mL M-CSF for 3 days. Cells remaining on the bottom of the wells were considered as bone marrow-derived macrophages and cultured in *α*-MEM containing 10% FBS, 50 ng/mL M-CSF, and 100 ng/mL RANKL. The culture medium was replaced with fresh medium every 3 days, and after 6 days, the cells differentiated into mature osteoclasts. The osteoclasts were used to investigate the formation of actin ring and to analyze the expression of regulating proteins.

#### 2.4.2. Assay for Formation of Actin Ring

 Osteoclastic cells (1 × 10^6^) induced with M-CSF and RANKL were seeded onto the 10 × 10 mm glass coverslips and treated with or without I, C, B, and their combination for 4 h. The osteoclasts were then fixed with 4% fresh paraformaldehyde in PBS for 15 min, washed three times with PBS, and then permeabilized with 0.1% Triton X-100 in PBS for 10 min. F-actin ring in the cells was labeled with rhodamine-conjugated phalloidin by incubating for 30 min in darkness. Nuclei were stained at 37°C for 10 min with 5 *μ*g/mL Hoechst 33258 solution, and after further washes in PBS, the cells were mounted in glycerol. Specimens were observed using a confocal laser scanning microscope (LEICA TCS-SP5, Germany) with appropriate combinations of filters and mirrors.

### 2.5. Western Blot

Cells from neonatal rat cavarial osteoblasts or osteoclasts induced with M-CSF and RANKL from bone marrow cells (as described in Section 2.4.1) were treated with or without I, C, B, and their combination for 24 h. Cells were lysed in a buffer containing 20 mM Tris–HCl, 150 mM NaCl, 1% Triton X-100, and protease and phosphatase inhibitors. The lysates (30–40 mg) were separated by 10% SDS PAGE and transferred to a polyvinylidene difluoride membrane. After blocking with 5% skim milk, the membrane was probed with anti-OPG and RANKL for osteoblasts or anti-phospho Akt, ERK, JNK, p38, and I*κ*-B for osteoclasts. The same membrane was stripped and reprobed and chemiluminescent signals were detected with a Gel Doc 2000 luminescent image analyzer (Wealtec Dolphin-Doc, USA).

### 2.6. Statistical Analysis

The experiments were repeated three times in five replicate samples. Data were expressed as mean ± standard deviation and one-way ANOVA, followed by Dunnett's *t*-test which was used for statistical analysis (PASW 18.0 software; SPSS Inc., Chicago, USA), and the level of significance was set at *P* < 0.05 (*), *P* < 0.01 (**), *P* < 0.001 (***). 

To determine if the compounds were acting synergistically, we used the probability sum test (*q* test) [19, 20]. The formula used is as follows: *q* = *E*_A+B_/(*E*_A_ + *E*_B_ − *E*_A_ × *E*_B_). Here, A and B indicate compound A and compound B;  *E* is the rate of change in the treated group compared with the mean values in the control group. *E*_A+B_ is the real percentage of responders and (*E*_A_ + *E*_B_ − *E*_A_ × *E*_B_) is the expected response rate. (*E*_A_ + *E*_B_) is the sum of the probabilities when compound A and compound B are used alone. (*E*_A_ × *E*_B_) is the probability of cells responding to both compounds when they were used alone. When *q* was <0.85, the combination was thought to be antagonistic; when *q* > 1.15, the combination was thought to be synergistic; when *q* was between 0.85 and 1.15, the combination was thought to be additive.

## 3. Results

### 3.1. ICB Decrease Bone Loss in Ovariectomized Osteoporotic Mice

As shown in Table 1, 12 weeks after ovariectomy, tibia bone mineral content (BMC) and bone mineral density (BMD) significantly decreased in total and trabecular bone compared with sham mice, but did not change in cortical bone. Administration of nylestriol significantly increased total and trabecular bone BMC and BMD in tibia compared to the OVX control. Administration of I, C, B, and their combination significantly increased BMC and BMD of trabecular bone and total BMD, but did not change total BMC in tibia. The effects of their combination were more potent than the individual compounds. These results indicate that I, C, B, and their combination improve BMC and BMD of trabecular bone and decrease bone loss induced by ovariectomy.

**Figure 1:**
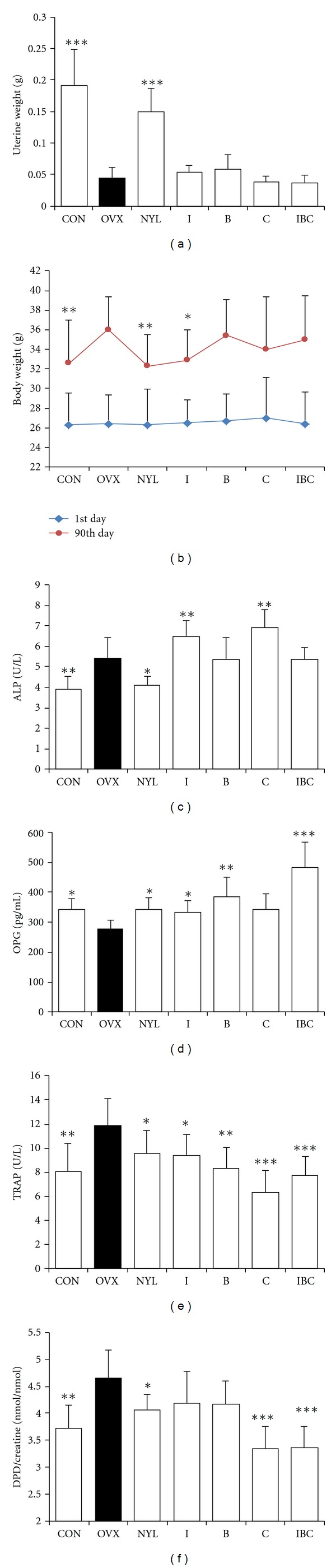
Effects of I, B, C, and their combination on ovariectomized osteoporotic mice. Ovariectomized mice were administered with I (40 mg/kg), B (120 mg/kg), C (20 mg/kg), and their combination (I 40 mg/kg + C 20 mg/kg + B 120 mg/kg) for 12 weeks. Then, the uterine and body weight were weighed, the serum biochemical parameters were assayed. (a) uterine weight; (b) body weight; (c) ALP activity; (d) OPG content; (e) TRAP activity; (f) DPD/creatine. Data were presented as mean ± standard deviation, (*n* = 10). *P < 0.05, and **P < 0.01, ***P < 0.001 compared with OVX control.

As shown in Figure 1, administration of I, C, B and their combination did not cause the increase of uterine weights and inhibit weight gain of ovariectomized mice, except for icariin which inhibited the body weights gain. Serum deoxypyridinoline cross-links to creatinine ratio (DPD/Cr), TRAP levels are biochemical markers of bone resorption, and ALP is a marker for bone formation. Ovariectomy induced high bone turnover in mice as evidenced by a significant increase in serum DPD/Cr, TRAP, and ALP levels. Nylestriol decreased serum DPD/Cr, TRAP, and ALP levels and inhibited high bone turnover in OVX mice. Administration of I, C, B, and their combination decreased serum DPD/Cr and TRAP levels, but no decrease in serum ALP levels. OPG (osteoprotegerin) is an endogenous protein produced by osteoblastic cells, which inhibits osteoclast formation and activation. Ovariectomy decreased serum OPG levels and in contrast, nylestriol increased serum OPG. The I, C, B, and their combination significantly increased OPG levels in ovariectomized mice. These results show that I, C, B, and their combination increased bone density by inhibiting bone resorption, and their combination showed higher activity than the individual compounds. Therefore, the effects and interaction of I, C, and B were further investigated for their mechanism of action and mutual interaction on osteoclastic bone resorption. 

### 3.2. ICB Synergistically Inhibits Osteoclast Formation and Differentiation

**Figure 2:**
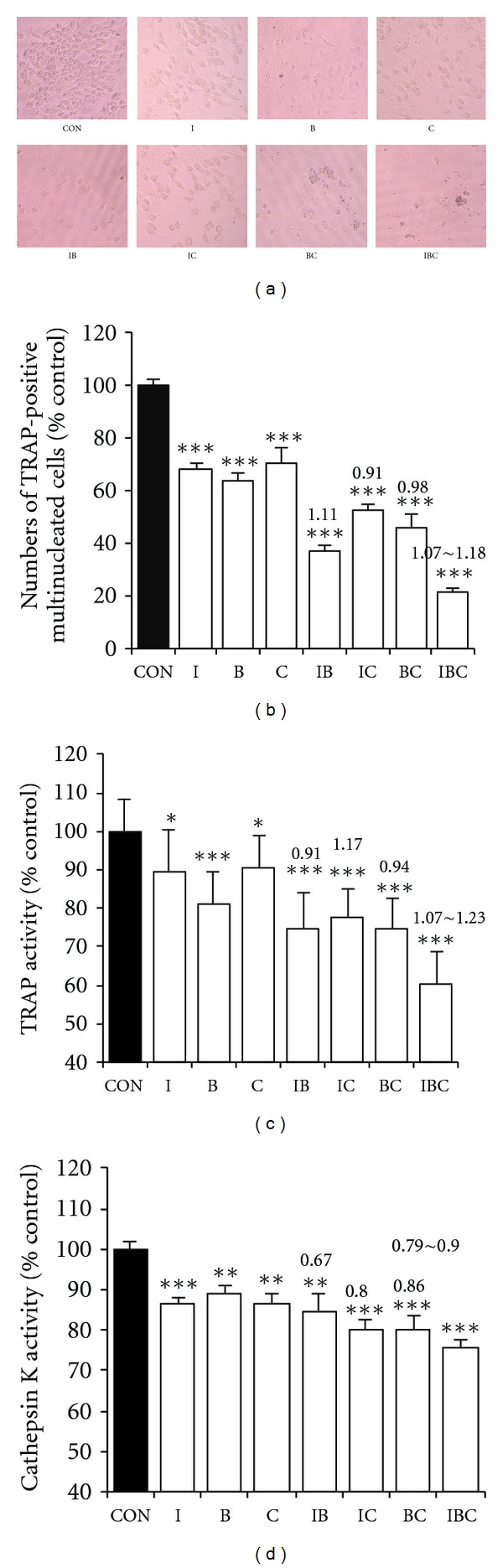
Inhibitory effects of I, B, C, and their combination on osteoclasts. (a) The morphological change of osteoclast (×200). Osteoclasts in the coculture system of primary osteoblasts and bone marrow cells were cultured for 24 h and then treated with or without the I, B, C, and their combination for 10 days. (b) Osteoclasts were treated for 10 days and then stained for TRAP, and those possessing three or more nuclei were counted as osteoclast-like multinucleated osteoclasts under a microscope. (c) Osteoclasts in the coculture system of primary osteoblasts and bone marrow cells were cultured for 6 days and then treated with or without I, B, C, and their combination for 48 hours. TRAP activity was measured by *p*-nitrophenyl sodium phosphate assay. (d) Cells were treated as described in TRAP assay, and the activity of cathepsin K was measured by a fluorometer equipped with a 400 nm excitation filter and 505 nm emission filter. Data were presented as mean ± standard deviation. The experiments were repeated 3 times in five replicate samples (*n* = 5) *P < 0.05, **P < 0.01, ***P < 0.001 compared with control. The number on the column is *q* value, indicating the interaction between I, B, and C.

To clarify the effect of I, C, and B alone or their combination on osteoclast formation, bone marrow cells were cocultured with osteoblasts derived from rat calvaria in the presence of 10^-8 ^M 1, 25-dihydroxyvitamin D_3_. Many TRAP-positive osteoclasts were formed in the coculture system within 6 days in response to 1*α*, 25(OH)_2 _D_3_. As shown in Figures 2(a) and 2(b), the numbers of TRAP-positive multinucleated cells were significantly reduced by I, C, and B or their combinations. In treatment of osteoclast with I, C, or B alone, the numbers of TRAP-positive multinucleated cells were reduced to 68.1%, 70.7%, and 63.5% of control, respectively. The combination of IB, IC, BC, and IBC decreased the number of osteoclasts to 37.1%, 53.0%, 46.0%, and 21.4% of control, respectively. According to the *q* value, the combination of IC, BC, and IB exerted additive inhibitory effects, and the combination of ICB exerted somewhat synergistic inhibitory effects on osteoclast formation. 

TRAP activity was directly related with osteoclastic bone resorption and as shown in Figure 2(c), after 48-h treatment, TRAP activity was significantly suppressed by I, B, C, and their combination. In treatment of osteoclast with I, B, and C alone, the TRAP activity decreased to 89.4%, 80.9% and 90.4% of control, respectively. The combination of IB, IC, BC, and IBC reduced the TRAP activity to 74.8%, 77.6%, 74.9%, and 60.2% of control, respectively. According to the *q* value, the combination of IB and BC exerted additive inhibitory effects; the combination of IC and ICB exerted synergistic inhibitory effects on the TRAP activity of osteoclast.

Another possible mechanism of the inhibitory effect on bone resorption of mature osteoclasts is that I, B, C, and their combination could reduce bone matrix degradation by inhibiting cathepsin K (CK) activity [21]; hence, we further evaluated the effects on cathepsin K activity of osteoclasts in a cell-free enzyme assay using synthetic substrate Ac-LR-AFC and recombinant cathepsin K. As shown in Figure 2(d), I, B, and C inhibited the osteoclastic CK activity to 86.4%, 88.9%, and 86.6% of control, respectively. Although the treatment of combinations of I, C, and B performed more potential suppressive effects than individual compounds, these combinations exerted antagonistic or additive, but not synergistic, inhibitory effects on cathepsin K activity of osteoclast according to the *q* value.

### 3.3. ICB Synergistically Suppresses Osteoclastic Bone Resorption

**Figure 3:**
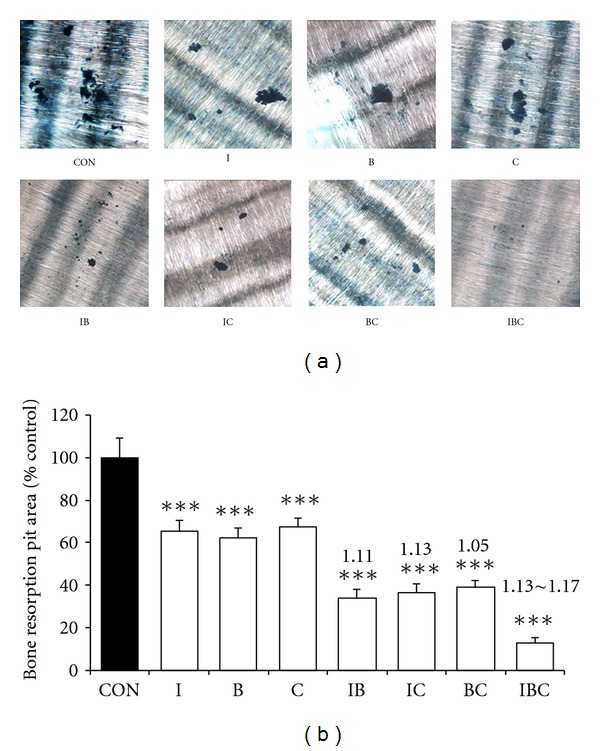
Inhibitory effects of I, B, C, and their combination on osteoclastic bone resorption. Primary osteoblasts and bone marrow cells were cocultured with a sterilized dental slice in *α*-MEM medium in the presence of 1, 25-dihydroxyvitamin D_3_ (10 nM) and dexamethasone (100 nM) and treated with or without I, B, C, and their combination for 12 days. Resorption pits were observed under a microscope, and the pit area was quantitated with image analysis software. (a) Bone resorption pit on dental slices (×100); (b) the change of bone resorption pit area under the treatment of I, B, C, and their combination. Data were presented as mean ± standard deviation. The experiments were repeated 3 times in five replicate samples (*n* = 5). *P < 0.05, **P < 0.01, ***P < 0.001 compared with control. The number on the column is *q* value, indicating the interaction between I, B, and C.

The dental slices were cocultured with osteoclast for 12 days. Untreated dental slices possess a very homogenous surface. Mature osteoclasts erode this homogenous surface and form resorption pits. After staining with toluidine blue, the resorption pits can be identified easily by their blue color. Under the experimental condition, a statistically significant reduction of number and area of bone resorption pit were observed on the dental slices treated with I, C, B, and their combination compared with control. In the mono-treatment group, I, C, or B alone decreased the area of bone resorption pit to 65.2%, 67.5%, and 62.4% of control. Bitreatment of I, C, and B decreased the area of bone resorption pit to 33.9%, 36.8%, and 39.4% of control. The ICB combination treatment decreased the area of bone resorption pit to 12.8% of control (Figures 3(a) and 3(b)). According to the *q* value, the combination of I, C, and B exerted slightly synergistic inhibitory effects on the bone resorption of osteoclast. These results suggested that ICB combination intensifies the inhibitory effect on osteoclastic bone resorption.

### 3.4. ICB Synergistically Blocks RANKL-Induced Osteoclasts Cytoskeletal Organization

**Figure 4:**
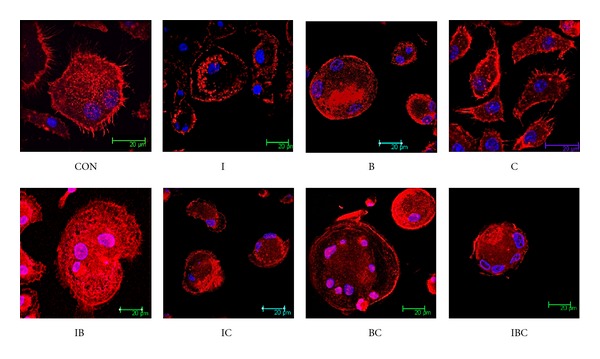
Effects of I, B, C, and their combination on F-actin rings. Osteoclasts were cultured on glass coverslip for 4 h in the absence (control) or presence of I, B, C, and their combination. The cells were then fixed and labeled with rhodamine-conjugated phalloidin and Hoechst 33258 and observed under confocal laser scanning microscope.

Osteoclastic bone resorption is initiated by the attachment of osteoclasts to the bone surface. Upon attachment, the osteoclasts form characteristic actin rings, a cytoskeletal structure essential for optimal osteoclastic bone resorption [22]. To determine if ICB affects cytoskeletal organization in osteoclasts, bone marrow cells were cultured on glass coverlips in the presence of M-CSF and RANKL with or without I, C, B, and their combination. The actin rings were visualized by staining with rhodamine-conjugated phalloidin to assess the activity in the early phase of bone resorption. As shown in Figure 4, confocal laser scanning microscopy revealed that actin rings were dense and had some pseudopodium lying in their periphery. Treatment of osteoclasts with I, C, B, and their combination caused pseudopodium to vanish; F-actin ring was disrupted and was loose and thin in the cytoplasmic area of osteoclasts. The effects of the combination of I, C, and B on the formation of F-actin ring are more potent than that of individual compounds, indicating that I, C, and B could synergistically affect cytoskeletal organization, which is necessary for bone resorption by osteoclasts.

### 3.5. ICB Elevates the Ratio of OPG and RANKL Expression in Osteoblast

**Figure 5:**
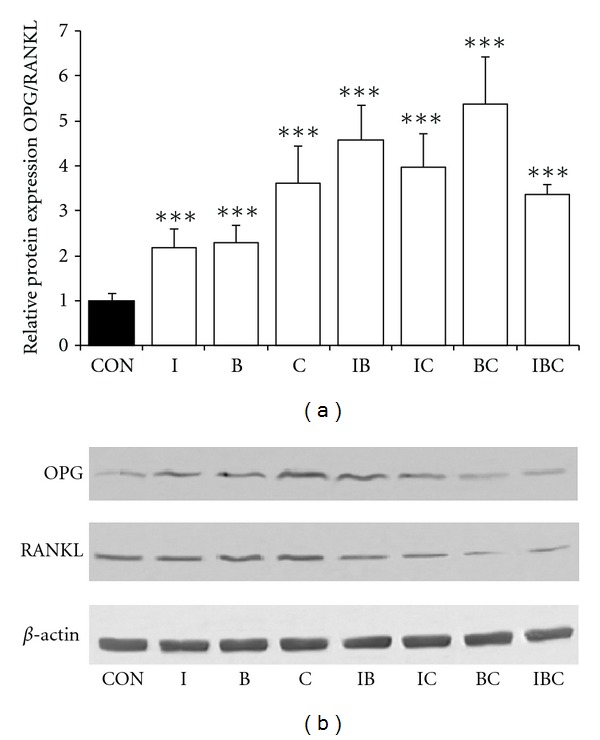
Effects of I, B, C, and their combination on expression of OPG and RANKL of osteoblasts. Primary osteoblastic cells from neonatal rat calvaria were treated with or without I, B, C, and their combination for 24 h. (a) The ratio of protein expression of OPG and RANKL. (b) Protein expression of OPG and RANKL was determined by Western blotting. *β*-actin was used as an internal reference. Data were presented as mean ± standard deviation. The experiments were repeated 3 times in five replicate samples (*n* = 5), *P < 0.05, **P < 0.01, ***P < 0.001 compared with control.

In coculture system, osteoblasts support the differentiation of osteoclast progenitors by expressing RANKL in response to vitamin D_3_ and dexamethasone [23]. Thus, it is necessary to determine whether these antiosteoclastogenic compounds were directly affecting osteoclast precursor cells or indirectly targeting to osteoblast and thus modulating the expression of OPG and RANKL, which are critical factors for osteoclast differentiation and activity. In treatment of osteoblast with I, C, B, and their combination for 24 h, the relative protein expressions of OPG in osteoblast were enhanced to 2.38-, 2.42-, 4.13-, 3.29-, 2.11-, 1.18-, and 1.32-fold of control, respectively; the relative protein expressions of RANKL were 1.09-, 1.06-, 1.14-, 0.72-, 0.53-, 0.22-, and 0.39-fold of control, respectively. Therefore, the ratios of the protein expression levels of OPG and RANKL were up to 2.17-, 2.29-, 3.62-, 4.56-, 3.98-, 5.37-, and 3.36-fold of control, respectively (Figure 5). These results indicated that I, B, C, and their combinations could regulate the OPG/RANKL system in osteoblast, resulting in the inhibition of osteoclast differentiation in a coculture model system.

### 3.6. ICB Causes the Decrease of Phosphorylation of RANK Signaling to MAPKs Pathways

**Figure 6:**
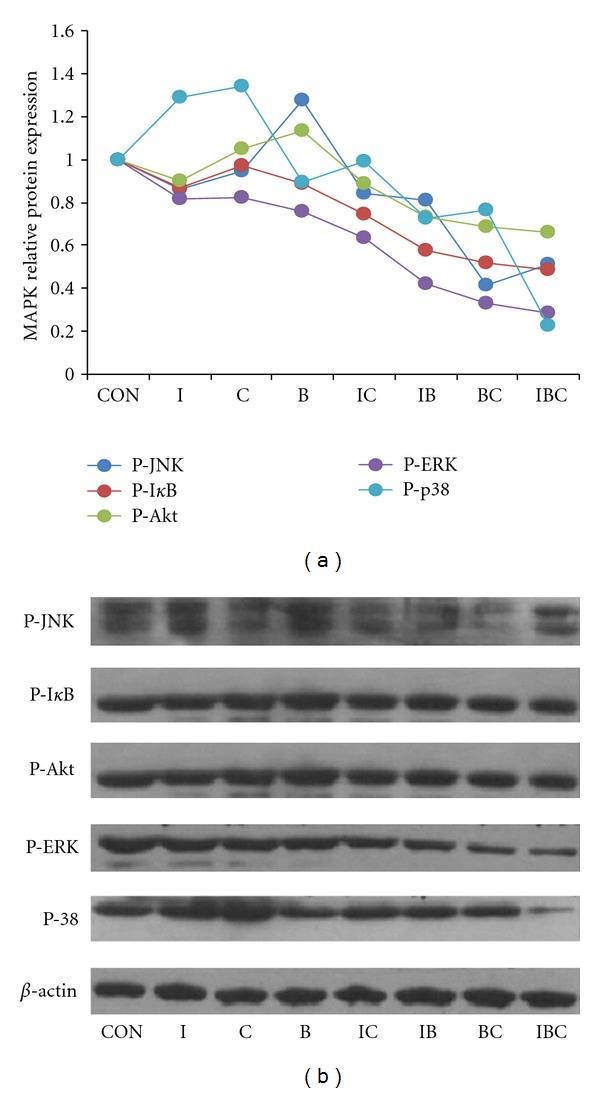
Effects of I, C, B, and their combination on RANKL-induced signaling pathways. Osteoclasts induced from bone marrow cells by M-CSF and RANKL were treated for 24 h and assessed for phosphorylation of JNK, ERK, p38 MAPK, and Akt and the degradation of I*κ*B*α* by Western blot. *β*-actin was used as an internal reference.

RANKL stimulation increases phosphorylation of Akt, ERK, JNK, and p38 in osteoclasts, and activation of the NF-*κ*B pathway requires the phosphorylation of I-*κ*B [24]. Thus, we next determined whether I, C, B, and their combination could affect the signaling pathways involving these kinases. As shown in Figure 6, treatment of osteoclasts with I, C, and B alone, the phosphorylation of I*κ*B and ERK were downregulated, and their combination showed more potent inhibitory effects than the individual compounds. Use of I and C alone upregulated the phosphorylation of p38, and the combination of IB, BC, and IBC significantly decreased this kinase; B increased the phosphorylation of Akt and JNK; all the combination of B with I and C reduced the phosphorylation of Akt and JNK. These results indicated that ICB synergistically decrease the phosphorylation of RANKL signaling in MAPK pathways, leading to the reduction of osteoclastic bone resorption. Taken together, our findings suggested the rationality of EXD in treating osteoporosis.

## 4. Discussions

In this study, we found that, in ovariectomized mice, combined use of the active components of EXD, namely, icariin (I), berberine (B), and curculigoside (C), significantly increased the bone mineral density and bone mineral content and regulated the serum biochemical parameters. At the cellular level, the IBC inhibited the formation, differentiation, and bone resorption of osteoclast through modulating OPG/RANKL system in osteoblast and MAPKs pathways in osteoclast. Assessment of the *q* value by the probability sum test directly demonstrated the synergic effect of I, B, and C on osteoclastic cells to some extent. These results clearly show that components of EXD exert effects of mutual reinforcement. However, IBC does not show an intensified adverse effect in the ovariectomized murine model, as revealed by change in body and uterine weight, consistent with the safety of EXD. These observations are in agreement with the rationality of the formula used in this study, mutual reinforcement of the compounds, and reduction of adverse effects.

Osteoclastic bone resorption is mediated by the formation of new osteoclasts and the bone-resorbing activity of osteoclasts. The mature osteoclasts are characterized by multinuclearity, TRAP staining, cathepsin K activity, an actin ring structure, ruffled border, and acidic cell condition during resorption [13]. As depicted in Figure 2, exposure of osteoclasts to I, B, C, and their combination significantly reduced the number of TRAP-positive multinucleated cells and the activity of TRAP and cathepsin K and induced the disruption of actin rings, indicating that I, B, C, and their combinations reduced the pit-forming activity of osteoclasts, both by triggering the direct disruption of actin rings and by inhibiting osteoclast formation and the survival of mature osteoclasts. Icariin, which exhibits the strongest therapeutic effects in the ovariectomized osteoporotic rats, has also been shown to be the principal ingredient of EXD in targeting osteoclastic bone resorption [16]; the B and C enhance the inhibitory effects of I on osteoclastic formation, differentiation, and bone resorption, augmenting the effects of I. Thus, the EXD formula proves its rational by modern biochemical analysis. 

The receptor activators of NF-*κ*B ligand (RANKL) and osteoprotegerin (OPG) synthesized by osteoblast plays an essential role in the osteoclastic formation, differentiation, and bone resorbing activity [7]. RANKL provides a signal to osteoclast progenitors through the receptor activator of NF-*κ*B (RANK) to activate osteoclast differentiation and function. OPG blocks the interaction between RANKL and the RANK receptor. In other words, OPG inhibits osteoclastogenesis while RANKL supports bone resorption of osteoclast [25]. Therefore, bone remodeling can be assessed by the relative ratio of OPG to RANKL. In the present study, I, B, C, and their combinations significantly increased the relative ratio of OPG to RANKL, indicating that they inhibited the bone resorption by modulating the expression of OPG and RANKL of osteoblast. 

The RANK signaling by the interaction with RANKL induces recruitment and activation of tumor necrosis factor receptor-associated factors (TRAFs), leading to the activation of MAPK pathway, including ERK, p38, and JNK [9]. The ERK pathway is involved in the negative regulation of osteoclastogenesis [6]. However, the p38 and JNK pathways have been shown to play a critical role during RANKL-induced osteoclast differentiation [26]. Osteoclast apoptosis is controlled by several signaling molecules, including TRAF6, Src, PI3 K/Akt, ERK, and NF-*κ*B pathways [27]. It was found in our study that the phosporylation of ERK, Akt, JNK, and I*κ*B was decreased slightly and that of osteoclast p38 was increased after I administration; the phosphorylation of ERK was downregulated after C or B administration alone; the phosphorylation of ERK, Akt, p38, JNK, and I-*κ*B of osteoclasts was downregulated significantly after combined administration of I with C or/and B. These results suggest that I, C, and B had different modulating mechanisms through MAPK pathways and their combinations had more potent inhibitory effects on the phosphorylation of these signaling protein of osteoclast.

It is worth pointing out that Chinese medicinal formula may have complicated changes by combining various herbs or their constituents. Some may reinforce or decrease their effects, moderate or eliminate their original toxic side effects; some may facilitate the delivery of the principle element to the disease site in the body [28]. In EXD, Epimedii Folium and Curculiginis Rhizoma have similar properties and effects their combination could reinforce each other's action. Phellodendri Chinensis Cortex has different pharmacological action from Epimedii Folium and Curculiginis Rhizoma but could assist in raising their therapeutic effects [17]. I, C, and B are major active constituents from Epimedii Folium, Curculiginis Rhizoma, and Phellodendri Chinensis Cortex, respectively. It may be an interesting observation in this work that ICB treatment decreases the osteoclastic bone resorption compared with I treatment alone, whereas I combined with C and/or B significantly upregulates OPG, and downregulates RANKL. Because OPG was upregulated, RANKL was downregulated 24 h after ICB treatment; some other events might also be involved in the enhancement of I action by C and B at the earlier stage. Interestingly, the same results were also observed in the MAPKs pathways. The phosporylation of P38, ERK, Akt, JNK, and I*κ*B was decreased slightly after I administration, while the phosphorylation of these signaling proteins was downregulated significantly after combined administration of I with C or/and B. These results suggest that C and B might assist I in the EXD formula to modulate these key pathways.

The antiosteoporotic activity and mechanism of icariin, curculigoside, and berberine have been extensively studied. Icariin enhances the differentiation and proliferation of osteoblasts and facilitates matrix calcification through the induction of BMP-2, BMP-4, and NO synthesis, subsequently regulating Cbf*α*1/Runx2, OPG, and RANKL gene expressions and activating BMP signaling [29–32]. Icariin also inhibited osteoclastic differentiation and reduced the motility and osteoclastic bone resorption [29, 33]. These regulatory effects of icariin on bone remodeling are also related with estrogen-like activity [30, 31]. Curculigoside has definite effects on osteoblast and osteoclast, enhances the expression of vascular endothelial growth factor and bone morphogenetic protein-2 in osteoblastic MC3T3, and prevents hydrogen peroxide-induced dysfunction and oxidative damage in calvaria osteoblasts [34–36]. Berberine inhibits RANKL-mediated osteoclast formation and survival through suppression of NF-*κ*B and Akt activation [37]. In osteoblastic cells, berberine enhanced the expression of osteogenic marker genes including osteopontin and osteocalcin through activation of Runx2 by p38 MAPK [38]. These findings indicated that icariin, curculigoside, and berberine modulate the bone metabolism through multitargets and pathways. Furthermore, EXD contains various kinds of active components, including flavonoids, phenolic glycoside, alkaloids, anthraquinone, organic acid, and polysaccharide. It is likely that a complicated interaction of active components exists on multiple targets. Therefore, the protective effects of EXD on bone loss are achieved in a similar manner in that multiactive components are exerting their effects through multitargets and pathways; however, these need to be investigated in more depth. 

In conclusion, the present study clarifies the interaction relationship on osteoclast between icariin, curculigoside, and berberine. This supports the theory that in TCM formula, the use of multiherbs or their ingredients can provide mutual reinforcement and assistance thus enhancing the therapeutic effects when compared to individual ingredients.

## Figures and Tables

**Table 1:** Effects of I, C, B, and their combination on bone mineral content (BMC) and bone mineral density (BMD) in OVX mice (*n* = 10).

Groups		BMC (mg/mm)			BMD (mg/cm^3^)	
	Total	Trabecular	Cortical	Total	Trabecular	Cortical
Sham control	1.77 ± 0.27	0.45 ± 0.13	0.89 ± 0.17	500 ± 23	220 ± 25	898 ± 19
OVX control	1.56 ± 0.13	0.33 ± 0.08^ΔΔΔ^	0.82 ± 0.13	426 ± 20^ΔΔΔ^	114 ± 16^ΔΔΔ^	910 ± 19
Nylestriol	1.86 ± 0.21*	0.43 ± 0.04**	0.78 ± 0.14	508 ± 29***	205 ± 20***	916 ± 28
OVX + I (40 mg/kg)	1.67 ± 0.09	0.40 ± 0.08**	0.81 ± 0.16	449 ± 31*	157 ± 25**	906 ± 20
OVX + C (20 mg/kg)	1.62 ± 0.07	0.37 ± 0.09*	0.79 ± 0.12	466 ± 34**	162 ± 30**	885 ± 19
OVX + B (120 mg/kg)	1.56 ± 0.12	0.38 ± 0.11*	0.86 ± 0.15	437 ± 45	149 ± 27**	881 ± 26
OVX + I (40 mg/kg) + C (20 mg/kg) + B (120 mg/kg)	1.52 ± 0.20	0.42 ± 0.05**	0.92 ± 0.17	482 ± 25**	195 ± 22**	889 ± 19

All values are expressed as mean ± SD, ^ΔΔΔ^ *P* < 0.001 versus sham group; *P < 0.05, **P < 0.01, ^∗ ∗ ∗^ *P* < 0.001 versus OVX group.
